# Surgical results of pelvic exenteration in the treatment of gynecologic cancer

**DOI:** 10.1186/1477-7819-12-279

**Published:** 2014-09-08

**Authors:** Andrea Petruzziello, William Kondo, Sergio B Hatschback, João A Guerreiro, Flávio Panegalli Filho, Cristiano Vendrame, Murilo Luz, Reitan Ribeiro

**Affiliations:** Surgical Oncology, Department of Surgery, Erasto Gaertner Hospital, Curitiba, Brazil; Clinical Research Department (CEPEP), Erasto Gaertner Hospital, Curitiba, Brazil; Department of Gynecology, VITA Batel Hospital, Curitiba, Brazil; Department of Gynecologic Oncology, Erasto Gaertner Hospital, Curitiba, Brazil; Department of Surgery, Erasto Gaertner Hospital, Curitiba, Brazil

**Keywords:** Complications, Pelvic exenteration, Uterine neoplasms

## Abstract

**Background:**

Our aim in the present study was to evaluate surgical outcomes and complications of pelvic exenteration in the treatment of gynecologic malignancy and to compare surgery-related complications associated with different types of exenteration.

**Methods:**

We performed a retrospective analysis of patients who underwent pelvic exenteration for the treatment of gynecologic cancer between January 2008 and August 2011. Patients were divided into two groups for comparison: total pelvic exenteration (TPE) and nontotal pelvic exenteration (NTE, including anterior pelvic exenteration (APE) posterior pelvic exenteration (PPE)). Outcomes are reported according to the modified Clavien-Dindo Classification of Surgical Complications.

**Results:**

Twenty-eight patients were included in the analysis. Eighteen had cervical cancer (64.3%). The prevalence of stage IIIB cervical cancer was 55%. Primary treatment with radiotherapy was performed in 53.3% of patients. Fifty percent of patients underwent TPE, 25% had APE and 25% underwent PPE. Patients who underwent TPE had worse outcomes, with a mean operative time of 367 minutes, use of blood transfusion in 93% of patients, ICU stay of 4.3 days and total hospital stay of 9.4 days. The overall mortality rate was 14.3%, and the surgical site infection rate was 25%. In the TPE group, 78.6% of patients experienced surgical complications. One-fourth of the total patient sample required reoperation, and the leading cause was urinary fistula (57.1%). Urinary leakage occurred in 22.7% of urinary reconstruction patients. Wet colostomy was the most common form of reconstruction with 10% of leakage.

**Conclusions:**

Postoperative urinary and infectious complications accounted for 75% of all causes of morbidity and mortality after pelvic exenteration. TPE is a more complex and morbid procedure than NTE.

## Background

In 1948, Brunschwig first described the pelvic exenteration (PE) procedure with purely palliative intent [1]. Over the next decades, in several reports of the use of this procedure as a curative treatment for pelvic or even perineal tumors, attempts were made to establish a more precise role for this operation [2–4].

The main indication for PE remains the treatment of recurrent or persistent cervical cancer previously treated with exclusive or concomitant chemoradiation [5]. PE in gynecologic cancer has also been well-described, although with less global experience, in the treatment of primary ovarian cancer [6] and recurrent endometrial cancer [7].

Those reports consistently describe high rates of surgery-related complications. High morbidity remains the most important barrier to the widespread use of this operation [8–13]. Additionally, few research groups were able to identify a specific risk category in which complications were more frequent. Furthermore, none of them suggested corrective strategies that could be implemented.

The main purpose of our present study was to investigate postoperative outcomes after PE for gynecologic cancer. We also tried to identify a subgroup of patients with a major risk for complications. We used the modified Clavien-Dindo Classification of Surgical Complications (Clavien-Dindo Classification) [14] for the first time in a PE series report, separately analyzing each type of PE: anterior (APE), posterior (PPE) and total (TPE).

## Methods

After obtaining the approval of the local research ethics committee of our institution, we reviewed and evaluated all medical records of patients who underwent PE for gynecologic cancer between January 2008 and August 2011 at our department. At the beginning of the procedures, the surgeons sought to identify intrapelvic unresectable disease and/or extrapelvic metastasis. Any tissues that raised suspicion of unresectability or metastasis were biopsied and sent for frozen sectioning to confirm unresectability.

For each patient, the following clinical variables were reviewed: age, tumor site and histology, clinical stage and previous therapy. For the analysis of intra- and postoperative outcomes, procedures were divided into APE (uterus plus bladder), PPE (uterus plus rectum) and TPE (uterus plus bladder and rectum). APE and PPE were analyzed separately and also grouped together as a “nontotal” pelvic exenteration group (NTE) and compared with TPE.

In each group, the following surgical variables are described: operative time (including both resection and reconstruction phases), amount of blood transfusion, time spent in the ICU and length of hospital stay, intraoperative complications, early postoperative complications (at 30 days), surgical site infection rate, need for reoperation and operative mortality. Complications were graded according to the Clavien-Dindo Classification [14]. Urinary reconstructions are described separately, including related complications and management strategies. Statistical analysis was carried out using SPSS v.13 software (SPSS, Chicago, IL, USA) and EPI Info 3.5 software (Centers for Disease Control and Prevention, Atlanta, GA, USA), applying the χ^2^ test, Fisher’s exact test and Student’s *t*-test when necessary.

## Results

### Demographics

We reviewed the cases of a total of 28 women who underwent PE between January 2008 and August 2011. Their mean age was 54.7 years (range, 34 to 78 years). The majority of patients presented with cervical cancer (64.3%). Ovarian and uterine cancer, as well as one patient with vaginal cancer, represented the remaining cases. In terms of cervical cancer, squamous cell carcinoma represented 83.3% of the sample. Among that subgroup, clinical stage IIIB (according to American Joint Committee on Cancer criteria) was the most common (44.4%), followed by stage IIB (22.2%).

Additional demographic characteristics are shown in Table 1, divided into surgical groups (APE, PPE and TPE). These groups were similar in their characteristics, except for the APE group, which showed significantly higher prevalence of previous radiotherapy (RT). However, this distinction was no longer present when we considered APE and PPE together as NTE.

**Table 1:** **Demographics** ^**a**^

	Overall	APE	PPE	TPE	***P***-value
Number of patients	28	7 (25%)	7 (25%)	14 (50%)	–
Mean age (range), yr	55 (24 to 78)	58 (43 to 78)	56 (36 to 73)	52 (34 to 73)	(0.725)
Site					
Cervical	18	6	3	9	(0.246)
Uterine	3	1	0	2	–
Ovarian	6	0	4	2	–
Vaginal	1	0	0	1	–
Clinical stage (cervical cancer)					
I	3	0	1	2	(0.246)
II	5	2	1	2	–
III	10	4	1	5	–
Previous radiotherapy^b^, *n* (%)	17 (61%)	7 (100%)	2 (28%)	8 (57%)	(0.021)

^a^APE, Anterior pelvic exenteration; PPE, Posterior pelvic exenteration; TPE = Total pelvic exenteration. ^b^ *P* = 0.698 for comparison of TPE with APE and PPE groups combined.

### Previous treatment

Preoperative treatment varied depending on the site of the disease. We observed that the majority of patients had previously been treated with a nonoperative approach. RT, either in combination with chemotherapy (CT) or alone, was the standard treatment in the majority of patients (53.6%), particularly in those with cervical cancer (83.3%). All the remaining patients had had previous surgical treatment, which was due to the fact that 32.1% of the sample was composed of patients with uterine or ovarian cancer, in which a primary surgical approach is usually the standard of care.

### Indication for the operation

In only three cases (one leiomyosarcoma of the uterine cervix and two ovarian cystadenocarcinomas) was a primary surgical approach with PPE performed. In all the remaining patients, the procedure was indicated for the treatment of recurrent or persistent disease.

### Type of procedure

According to type of exenteration, TPE accounted for the majority of cases (50%), followed by APE (25%) and PPE (25%).

### Surgical outcomes

Detailed data on each type of PE are shown in Table 2. Mean operative time for TPE was 367 minutes (range, 240 to 540 minutes), which is longer than for NTE (271 minutes) (*P* = 0.02).

**Table 2:** **Surgical outcomes** ^**a**^

	Overall	APE	PPE	TPE	NTE	***P***-value
Number of patients	28	7	7	14	14	–
Mean operating time (min)	269	310	232	*367*	*271*	*0.023*
Blood transfusion required, *n* (%)	22 (78.6%)	5 (71.4%)	4 (57.1%)	13 (92.8%)	9 (64.3%)	0.082
Mean blood transfusion (units)	2.2	3.1	0.8	2.6	1.9	0.456
Mean stay ICU (days)	3.2	2.1	2.1	4.3	2.1	0.138
Mean total hospital stay (days)	9.3	6.3	8.7	11.1	7.5	0.129
Surgical site infection, n (%)	7 (25%)	1 (14.3%)	0	*6 (42.8%)*	*1 (7.1%)*	*0.038*
Need for reoperation, n (%)	7 (25%)	2 (28.6%)	0	5 (35.7%)	2 (14.2%)	0.192
Perioperative mortality, n (%)	4 (14.3%)	1 (14.3%)	0	3 (21.4%)	1 (7.1%)	0.297

^a^APE, Anterior pelvic exenteration; NTE, Nontotal exenteration; PPE, Posterior pelvic exenteration; TPE, Total pelvic exenteration. *P*-value is for comparison between TPE and NTE, and *P* < 0.05 was set as the level of significance.

Other surgical outcomes showed worse results for TPE, such as need for blood transfusion (93%), mean units of packed red cell blood cells transfused (2.6), length of ICU stay (4.3 days, ranging from 2 to 21) and hospital stay (11.1 days, ranging from 5 to 24). However, these results were not statistically significant.

### Morbidity and mortality

There were major intraoperative complications in only four patients: one case of accidental bladder injury, one case of hemorrhagic shock due to vascular injury to iliac vessels and two cases of uncontrolled fecal spillage into the abdominal cavity. No intraoperative deaths occurred.

Overall perioperative mortality was 14.3%. The mortality rate of TPE patients was 21.4%, and that of APE patients was 14.3%. No perioperative deaths occurred in the PPE group. There was no statistically significant difference in mortality between the TPE and NTE groups.

**Figure 1:**
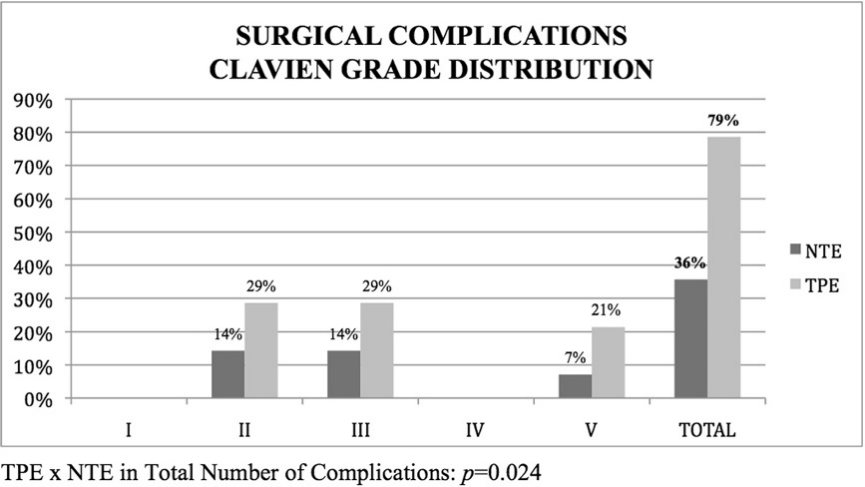
**Distribution of surgical complications.** NTE, Nontotal exenteration; TPE, Total pelvic exenteration.

The analysis of the early postoperative complications (at 30 days) was carried out according to the Clavien-Dindo Classification. Postoperative complications were more frequent and more severe in the TPE group than in the NTE group; 78.6% of TPE patients experienced some type of complication compared to 35.7% in the NTE group (*P* = 0.02). Table 3 and Figure 1 present the distribution and severity of complications as measured according to the Clavien-Dindo Classification. The overall surgical site infection rate was 25%; in the individual groups, the rates were 14.3% in the APE group, 0% in the PPE group and 42.8% in the TPE group (*P* = 0.03).

**Table 3:** **Surgical complications grade (Clavien-Dindo Classification)** ^**a**^

	Overall	APE	PPE	TPE	NTE	***P***-value
Number of patients	16/28 (57.1%)	3/7 (42.8%)	2/7 (28.6%)	11/14 (78.6%)	5/14 (35.7%)	0.024
I	0	0	0	0	0	–
II	6 (21.4%)	1 (14.3%)	1 (14.3%)	4 (28.6%)	2 (14.3%)	0.324
III	6 (21.4%)	1 (14.3%)	1 (14.3%)	4 (28.6%)	2 (14.3%)	0.324
IV	0	0	0	0	0	–
V	4 (14.3%)	1 (14.3%)	0	3 (21.4%)	1 (7.1%)	0.297

^a^APE, Anterior pelvic exenteration; NTE, Nontotal exenteration; PPE, Posterior pelvic exenteration; TPE, Total pelvic exenteration. *P*-value is for comparison between TPE and NTE, and *P* < 0.05 was set as the level of significance.

### Need for reoperation

One-fourth of patients required some form of surgical reoperation. However, reoperations were more common in patients who underwent TPE (35.7%) than in the APE patients (28.6%). PPE patients required no further intervention. There was no statistical difference in the rate of reoperations. The most common reason for reoperation was urinary fistula (57.1%) that was not manageable with clinical or percutaneous treatment. The remaining cases occurred because of evisceration along with internal hernia (one case) and small- bowel obstruction (two cases).

### Urinary tract reconstruction and complications

The most common type of urinary reconstruction was wet colostomy (ten cases), followed by ureterostomy (four cases) and ileal conduit (three cases). The overall leakage rate was 22.7%. Leakages were managed successfully with conservative treatment in 40% of the cases. The remaining patients required reoperation. The types of reconstruction and complications are detailed in Table 4.

**Table 4:** **Urinary tract reconstruction and fistulas**

Reconstruction type	Number of patients in each group	Fistulas, ***n***(%)
Wet colostomy	10	1 (10%)
Ileal conduit	3	1 (33.3%)
Segmental resection of the ureter	1	1 (100%)
Ureteral reimplant	2	2 (100%)
Nephrostomy	2	0
Ureterostomy	4	0
Overall	22	5 (22.7%)

### Colon reconstruction and complications

Excluding the 10 cases of wet colostomy, the other 11 patients required some form of colon reconstruction. Terminal colostomy was performed in seven patients and primary anastomosis in four; only one patient received a protective stoma, a colostomy. As a routine, we perform radiological control of the anastomosis only in cases of a clinically apparent fistula. In the four patients with colonic anastomosis, no clinically evident leakage or related complications occurred.

### Neovagina

In the only attempt of genital reconstruction with neovagina, which was performed in a 45-year-old patient with central recurrence of an irradiated stage IIB cervical squamous cell carcinoma requiring TPE, partial necrosis of the flap (gracilis muscle) occurred, resulting in sepsis and a need for surgical debridement.

### Margin status after operation

A complete tumor resection with negative margins was achieved in 92.8% of the operations, microscopically involved margins in 4.3% (one case) and macroscopic residual disease in 4.3% (one case). In the latter case, at the end of the operation, a small but nonresectable node at the root of the mesentery was detected.

### Adjuvant therapy

Most of the patients (78.6%) did not receive any adjuvant treatment.

## Discussion

Our institution is a tertiary referral cancer center in the south of Brazil, in which approximately 5,000 new patients with confirmed malignant neoplasia are treated annually. On average, nearly 300 of them present with a diagnosis of cervical carcinoma, and 84% of cases are classified as stage II, III or IV [15].

Squamous cell carcinoma of the uterine cervix is the most common gynecologic malignancy in developing countries, including Brazil. In Brazil, most of the patients present upon diagnosis with locally advanced disease [16]. In our institution, the typical patient candidate for possible PE is a woman with recurrent cervical carcinoma who has previously received primary treatment with RT (pelvic RT plus brachytherapy). In our present case review, this group comprised 57.1% of the cases. Many of those cervical cancer patients already had locally advanced severe disease classified as clinical stage IIIB at the time of the initial diagnosis (50% in our cohort). These population characteristics differ somehow from those reported in the United States and Europe, leading us to suppose that we are dealing with patients in worse clinical condition.

PE remains the only curative procedure for patients with recurrent cervical cancer after nonsurgical treatment (RT with or without CT). Another condition where PE is appropriate is primary ovarian cancer in which resection of the bladder and/or rectum is necessary to achieve free margins or optimal cytoreduction [6]. Also, uterine cancer sometimes presents as a locally recurrent neoplasia in which some irradiation of the pelvis has already been performed as part of the primary treatment and for which a radical surgical approach is necessary to pursue a curative treatment [7]. Our sample also included one case of uterine sarcoma.

The main purpose of this retrospective review was to analyze surgical complications by comparing different types of PE. We do not report survival analysis, given the lack of follow-up time to date. TPE was the most commonly performed operation, possibly because of recurrence severity due to more advanced disease at diagnosis.

The Clavien-Dindo Classification of surgical complication was used. On the basis of that system, the results suggest not only that surgical complications are high in incidence but also that they are severe in most cases.

After analyzing the profile of postoperative outcomes, it seems that TPE is a procedure very distinct from APE and PPE in terms of surgical complexity, operative time, recovery time and, particularly, the rate and severity of surgery-related complications. In our study, TPE led to a statistically significant higher incidence of complications. We could not demonstrate that those complications were more severe than the complications that occurred in the NTE group, possibly because of the small sample size. However, there was a clear trend for worse complications in the TPE group in every single grade according to Clavien-Dindo Classification.

Urinary diversion and reconstruction remains a serious issue in patients who undergo PE [17]. Most of them had previously received pelvic irradiation with the risk of chronic vascular alteration of the small bowel and urinary tract, mainly fibrosis and sclerosis of the small vessels. Any kind of anastomosis using this poorly vascularized tissue presents a high risk of leakage. We found significant rates of complications after urinary reconstruction, higher than is the rates reported by many North American and European centers.

It is common in our public hospital setting that patients still receive two-dimensional irradiation, with low utilization of techniques such as three-dimensional irradiation, intensity-modulated RT and image-guided RT. It has been demonstrated that those techniques can help in lowering the radiation dose received by the small bowel, particularly in the setting of extended-field RT [18, 19]. In our experience, the ileum quality and vascularization were frequently impaired, as was the distal portion of ureters. After an initial bad experience with ileal conduits, we found wet colostomy to be a safer option and an attractive alternative for patients treated with TPE.

On the basis of our results, we can explain some of the apparent differences between the APE and PPE groups. When PPE is performed, the urinary tract is rarely manipulated, leading to a reduced chance of postoperative complication. We did not make a direct comparison between those patients in our study, owing to the limited size of both groups.

We report similar postoperative morbidity but a higher mortality rate (14.3%) compared to other published series of PE patients. Although an exact and unique explanation for these results is difficult to find in our small number of cases, some hypotheses and suggestions for improvement can be offered.

Treatment protocols were not standardized during our early years of experience, and, for that reason, several modifications were implemented over time. The use of wet colostomy as a urinary and fecal reconstruction method after TPE seems to be a safer option, with some authors suggesting that it is the best option in this scenario [20–22]. A more systematic use of this reconstruction technique could lead to a reduction in urinary diversion complications and operative mortality.

With widespread use of more modern RT techniques, such as three-dimensional irradiation, in our public system, we may expect fewer bowel-related complications and deal with a better-vascularized ileum and terminal ureter. Centralizing surgical expertise can also be an effective strategy to improve experience in uncommon procedures such as PE.

An inferior overall clinical condition and lower socioeconomic status of the patients [23] may also have played a role in our results.

### Limitations

Major biases of our study are the retrospective nature of the analysis and the limited number of patients. These factors are present in the majority of series reported in the literature on PE. To date, only one study group has prospectively analyzed surgical and survival outcomes of this procedure [10]. Possible reasons for this are the rarity of this operation in North America and Europe and that there are only limited reports on this procedure from countries that actually perform it in a more constant and routine fashion, such as Brazil and countries in Africa and Asia.

## Conclusion

PE for the treatment of gynecologic malignancy is an aggressive choice. The initial experience in our institution shows high rates of morbidity and mortality, although these rates are comparable to those of other series described in the literature. Thus, patient selection must be accurate, and it is advisable to perform this procedure only in high-volume cancer centers with experience in complex pelvic and abdominal surgery.

Major complications remain secondary to urinary reconstruction and infection. These are frequently severe and usually require reoperation.

Patients requiring TPE seem to be part of a high-risk subgroup with more challenging operations and worse postoperative outcomes.

It is worth emphasizing that PE, although it is a high-risk procedure, is the only curative option for these patients. In countries such as Brazil, the development of experience in this field is fundamental because of the endemic behavior of this neoplasia, particularly in advanced stages.

## Abbreviations

APE: Anterior pelvic exenteration
CT: Chemotherapy
NTE: Nontotal pelvic exenteration
PE: Pelvic exenteration
PPE: Posterior pelvic exenteration
RT: Radiotherapy
TPE: Total pelvic exenteration.

## References

[1] Brunschwig A (1948). Complete excision of pelvic viscera for advanced carcinoma; a one- stage abdominoperineal operation with end colostomy and bilateral ureteral implantation into the colon above the colostomy. Cancer.

[2] Phillips B, Buchsbaum HJ, Lifshitz S (1981). Pelvic exenteration for vulvovaginal carcinoma. Am J Obstet Gynecol.

[3] Shepherd JH, Ngan HY, Neven P, Fryatt I, Woodhouse CRJ, Hendry WF (1994). Multivariate analysis of factors affecting survival in pelvic exenteration. Int J Gynecol Cancer.

[4] Berek JS, Howe C, Lagasse LD, Hacker NF (2005). Pelvic exenteration for recurrent gynecologic malignancy: survival and morbidity analysis of the 45-year experience at UCLA. Gynecol Oncol.

[5] Maggioni A, Roviglione G, Landoni F, Zanagnolo V, Peiretti M, Colombo N, Bocciolone L, Biffi R, Minig L, Morrow CP (2009). Pelvic exenteration: ten-year experience at the European Institute of Oncology in Milan. Gynecol Oncol.

[6] Tixier H, Fraisse J, Chauffert B, Mayer F, Causeret S, Loustalot C, Deville C, Bonnetain F, Sagot P, Douvier S, Cuisenier J (2010). Evaluation of pelvic posterior exenteration in the management of advanced-stage ovarian cancer. Arch Gynecol Obstet.

[7] Khoury-Collado F, Einstein MH, Bochner B, Alektiar KM, Sonoda Y, Abu-Rustum NR, Brown CL, Gardner GJ, Barakat RR, Chi DS (2012). Pelvic exenteration with curative intent for recurrent uterine malignancies. Gynecol Oncol.

[8] Goldberg GL, Sukumvanich P, Einstein MH, Smith HO, Anderson PS, Fields AL (2006). Total pelvic exenteration: the Albert Einstein College of Medicine/Montefiore Medical Center experience (1987 to 2003). Gynecol Oncol.

[9] Ungar L, Palfalvi L, Novak Z (2008). Primary pelvic exenteration in cervical cancer patients. Gynecol Oncol.

[10] Park JY, Choi HJ, Jeong SY, Chung J, Park JK, Park SY (2007). The role of pelvic exenteration and reconstruction for treatment of advanced or recurrent gynecologic malignancies: analysis of risk factors predicting recurrence and survival. J Surg Oncol.

[11] Fotopoulou C, Neumann U, Kraetschell R, Schefold JC, Weidemann H, Lichtenegger W, Sehouli J (2010). Long-term clinical outcome of pelvic exenteration in patients with advanced gynecological malignancies. J Surg Oncol.

[12] Jurado M, Alcázar JL, Martinez-Monge R (2010). Resectability rates of previously irradiated recurrent cervical cancer (PIRCC) treated with pelvic exenteration: Is still the clinical involvement of the pelvis wall a real contraindication? A twenty-year experience. Gynecol Oncol.

[13] Benn T, Brooks RA, Zhang Q, Powell MA, Thaker PH, Mutch DG, Zighelboim I (2011). Pelvic exenteration in gynecologic oncology: a single institution study over 20 years. Gynecol Oncol.

[14] Dindo D, Demartines N, Clavien PA (2004). Classification of surgical complications: a new proposal with evaluation in a cohort of 6336 patients and results of a survey. Ann Surg.

[15] **Relatório RHC 2005–2009** [http://www.erastogaertner.com.br/arquivos/rhc/RelatorioRHC2005a2009.pdf]

[16] Instituto Nacional de Câncer José Alencar Gomes da Silva (INCA): **Estimativa 2012: Incidência de Câncer no Brasil.** [http://portal.saude.sp.gov.br/resources/ses/perfil/gestor/homepage/estimativas-deincidencia-de-cancer-2012/estimativas_incidencia_cancer_2012.pdf]

[17] Bladou F, Houvenaeghel G, Delpéro JR, Guérinel G (1995). Incidence and management of major urinary complications after pelvic exenteration for gynecological malignancies. J Surg Oncol.

[18] Jadon R, Pembroke CA, Hanna CL, Palaniappan N, Evans M, Cleves AE, Staffurth J (2014). A systematic review of organ motion and image-guided strategies in external beam radiotherapy for cervical cancer. Clin Oncol (R Coll Radiol).

[19] Portelance L, Clifford Chao KS, Grigsby PW, Bennet H, Low D (2001). Intensity-modulated radiation therapy (IMRT) reduces small bowel, rectum, and bladder doses in patients with cervical cancer receiving pelvic and para-aortic irradiation. Int J Radiat Oncol Biol Phys.

[20] Backes FJ, Tierney BJ, Eisenhauer EL, Bahnson RR, Cohn DE, Fowler JM (2013). Complications after double-barreled wet colostomy compared to separate urinary and fecal diversion during pelvic exenteration: time to change back?. Gynecol Oncol.

[21] Guimaraes GC, Ferreira FO, Rossi BM, Aguiar S, Zequi SC, Bachega W, Nakagawa WT, Fonesca FP, Sarkis AS, Lopes A (2006). Double-barreled wet colostomy is a safe option for simultaneous urinary and fecal diversion: analysis of 56 procedures from a single institution. J Surg Oncol.

[22] Golda T, Biondo S, Kreisler E, Frago R, Fraccalvieri D, Millan M (2010). Follow-up of double-barreled wet colostomy after pelvic exenteration at a single institution. Dis Colon Rectum.

[23] da Fonseca AJ, Ferreira LP, Dalla-Benetta AC, Roldan CN, Ferreira ML (2010). Epidemiology and economic impact of cervical cancer in Roraima, a northern state of Brazil: the public health system perspective (Article in Portuguese). Rev Bras Ginecol Obstet.

